# Number Line Estimation Predicts Mathematical Skills: Difference in Grades 2 and 4

**DOI:** 10.3389/fpsyg.2017.01576

**Published:** 2017-09-12

**Authors:** Meixia Zhu, Dan Cai, Ada W. S. Leung

**Affiliations:** ^1^Department of Psychology, Shanghai Normal University Shanghai, China; ^2^Faculty of Education, Qingdao Binhai University Qingdao, China; ^3^Department of Occupational Therapy, Neuroscience and Mental Health Institute, University of Alberta, Edmonton AB, Canada

**Keywords:** number line estimation, calculation fluency, math problem-solving, general cognitive abilities, Chinese children

## Abstract

Studies have shown that number line estimation is important for learning. However, it is yet unclear if number line estimation predicts different mathematical skills in different grades after controlling for age, non-verbal cognitive ability, attention, and working memory. The purpose of this study was to examine the role of number line estimation on two mathematical skills (calculation fluency and math problem-solving) in grade 2 and grade 4. One hundred and forty-eight children from Shanghai, China were assessed on measures of number line estimation, non-verbal cognitive ability (non-verbal matrices), working memory (N-back), attention (expressive attention), and mathematical skills (calculation fluency and math problem-solving). The results showed that in grade 2, number line estimation correlated significantly with calculation fluency (*r* = -0.27, *p* < 0.05) and math problem-solving (*r* = -0.52, *p* < 0.01). In grade 4, number line estimation correlated significantly with math problem-solving (*r* = -0.38, *p* < 0.01), but not with calculation fluency. Regression analyses indicated that in grade 2, number line estimation accounted for unique variance in math problem-solving (12.0%) and calculation fluency (4.0%) after controlling for the effects of age, non-verbal cognitive ability, attention, and working memory. In grade 4, number line estimation accounted for unique variance in math problem-solving (9.0%) but not in calculation fluency. These findings suggested that number line estimation had an important role in math problem-solving for both grades 2 and 4 children and in calculation fluency for grade 2 children. We concluded that number line estimation could be a useful indicator for teachers to identify and improve children’s mathematical skills.

## Introduction

Mathematical competence comprises a wide variety of different cognitive processes such as working memory (Fuchs et al., 2010a,b; Swanson, 2011; Traff, 2013), attention (Kercood et al., 2004; Anobile et al., 2013; Mahmoudi et al., 2015), and estimation skills (Siegler and Booth, 2004; Booth and Siegler, 2006, 2008; Halberda et al., 2008; Schneider et al., 2009; Cohen and Sarnecka, 2014). Researchers have found that numerical abilities correlate with cognitive processes (Fuchs et al., 2010a,b; Traff, 2013). Children with strong estimation skills have greater working memory capacity, better counting and arithmetic skills, and higher math achievement test scores than children who estimate less accurately (Newman and Berger, 1984; LeFevre et al., 1993; Siegler and Booth, 2004). Numerical estimation refers to the skill in which people, instead of counting objects one by one in the field of vision, adopt the way of estimation for getting an approximate number (Booth and Siegler, 2006). Number line estimation is part of numerical estimation and is important for developing the sense of numbers in children (Dehaene, 1997). Number line estimation is the ability to place numbers on a physical line and has closely relationship with analogical reasoning skills when estimating (Sullivan and Barner, 2014). Number line estimation has been found to correlate strongly with math problem-solving (Berch, 2005; Locuniak and Jordan, 2007; Halberda and Feigenson, 2008). In addition, Starr et al. (2013) have shown that number sense in preschool are related to mathematical abilities in childhood, even after controlling for intelligence quotient (IQ). Specifically, many studies have demonstrated that number line estimation is an important predictor of math achievement in young children as well as older children (fifth and sixth graders) and a predictor of further achievement (Siegler and Booth, 2004; Booth and Siegler, 2006; Geary et al., 2009; Schneider et al., 2009). Booth and Siegler (2006) have shown that number line estimation correlates positively with math achievement from kindergarten to third grade. Their results indicated that children making smaller number line estimation errors had higher math achievement test scores. Following this line of thought, number line estimation would be a legitimate tool for developing mathematics ability in children. In fact, many researches have suggested that number line estimation is an important predictor of the development of mathematical ability in young children (Siegler and Booth, 2004; Booth and Siegler, 2006; Halberda et al., 2008; Schneider et al., 2009; Cohen and Sarnecka, 2014). Specifically, Booth and Siegler (2006) have reported that all types of estimation skills are positively related to math achievements.

Approximate number system (ANS) refers to a cognitive processing system, which is independent of external cues such as words or instructions, for estimating the magnitude of a group of numbers. With ANS, representation grows with the target numerosity, producing inexact number representations (Halberda and Feigenson, 2008). The ANS is activated when adults perform symbolic number tasks (Piazza et al., 2007) and may even provide a foundation for more sophisticated mathematics (Gilmore et al., 2007). Number line estimation is a part of pure numerical estimation that has a goal of approximating some quantitative value (Booth and Siegler, 2006). It means that when the person perform the number line estimation task, the ANS will be activated. Halberda et al.’s (2008) speculated that ANS serves as the cognitive basis for symbolic mathematics and it may provide a foundation for more sophisticated mathematics (Gilmore et al., 2007). Some studies found that cognition was related with number line estimation (Jordan et al., 2013; Namkung and Fuchs, 2016). Namkung and Fuchs (2016) found that non-verbal reasoning and numerical working memory significantly predicted whole-number number line estimation. Jordan et al.’s (2013) also found that non-verbal reasoning, attention, and working memory significantly predicted whole-number number line estimation skills.

However, past studies relating number line estimation and math achievements have not taken into account other key predictors of mathematical abilities. In Schneider et al.’s (2009) study, number line estimation correlated significantly with math achievement but that study had not addressed how age, non-verbal cognitive ability, attention, and working memory might have affected the findings. The importance of cognitive abilities on number line estimation has been established. For example, recent studies have shown that number line estimation depends not only on number knowledge, but also on other domain-general abilities like cognitive abilities such as attending, memorizing, and other executive functions (skills that are not directly related to numbers; Barth and Paladino, 2011; Anobile et al., 2013). Other studies (e.g., Geary, 2004; Passolunghi and Pazzaglia, 2004; Andersson, 2007; Berg, 2008) have suggested that number line estimation may rely on cognitive abilities such as working memory (Andersson, 2007; Bull et al., 2008) and attention (Kercood et al., 2004; Anobile et al., 2013; Mahmoudi et al., 2015). Among them, attention has been shown crucial for math achievement in primary school children (Kercood et al., 2004; Mahmoudi et al., 2015). Recently, Fuchs et al. (2010a,b) controlled for general cognitive abilities (e.g., working memory, executive functions, language, concept formation, and non-verbal problem solving) and found that number line estimation accounted for individual differences in word problem-solving skill. Therefore, including cognitive abilities in studying the relationship between number line estimation and math achievements would provide a more comprehensive understanding of the role of number line estimation on mathematic skills.

Despite the omnipresence and importance of estimation in children’s lives, far less is known about the effect of number line estimation on different mathematical skills in different grades after controlling for age and other cognitive processes. Two fundamental mathematical skills are calculation fluency and math problem-solving (Cai et al., 2016). Calculation fluency refers to simple mathematical task in which students are asked to extract the relevant rules and information from long-term memory (Kroesbergen et al., 2010), without multi-step operation. Math problem solving task involves multiple cognitive processes, including problem representation and problem execution. Problem representation requires students to understand and integrate relevant information, maintain the psychological representation of the problem in working memory, find the path to problem solving, and ultimately form a consistent problem solution and then problem execution proceed to monitor and debug in the process (Li, 2009; Li and Wang, 2010). Fuchs et al. (2010a,b) and Geary (2011) have suggested that general cognitive abilities and the ability to exactly and approximately estimate numbers are uniquely foundational to mathematical ability in early childhood. Indeed, cognitive processes have been found to be strong predictors of math achievements including calculation fluency and math problem solving (Berg, 2008; Bull et al., 2008; Geary, 2011; Anobile et al., 2013). However, different cognitive processes seem to have differential impact on mathematical skills. Past studies have shown that attention has an independent predictive effect on mathematical calculation in junior students (Naglieri and Rojahn, 2004; Kroesbergen et al., 2010). With the increase in grade, the effect of attention gradually diminishes, and planning and problem solving skills has a greater predictive effect than attention on mathematical calculation (Naglieri and Rojahn, 2004; Kroesbergen et al., 2010). On the other hand, non-verbal cognitive ability has been shown to play a significant role in math problem solving (Kroesbergen et al., 2010). As the development of students’ cognitive ability and the demands for knowledge about mathematical calculation increases, non-verbal cognitive ability is expected to play an important role in middle and high grades. In line of this thought, non-verbal cognitive ability would have a more pronounced effect on complex mathematical tasks in middle and high grades, and attention would have a significant effect on simple mathematical tasks in lower grades.

In the present study, we examined the contribution of number line estimation to two different mathematical skills (calculation fluency and math problem-solving). There were two objectives. First, we examined the role of number line estimation on mathematical skills after controlling for age, non-verbal cognitive ability, attention, and working memory. Taking into account the effects of cognitive processes provides a conservative test for understanding the role of number line estimation in different aspects of mathematical skills (calculation fluency and math problem-solving). Second, we examined how number line estimation predicts calculation fluency and math problem-solving in different grades (grades 2 and 4) in China.

## Materials and Methods

### Participants

One hundred and forty-eight local children were recruited from two public elementary schools in Shanghai, China, which were located in different parts of the city in order to increase our chances to include children of various demographics. Among them, 77 were second graders (33 females and 44 males; mean age = 92.65 months, *SD* = 3.93) and 71 were fourth graders (29 females and 42 males; mean age = 116.28 months, *SD* = 3.50). Participation was completely voluntary and provided written informed consent. Each child received some stickers or notebooks as reward at the end of the tests. All children were native Mandarin speakers and none of them was diagnosed with intellectual, sensory, or behavioral disorders. All children received mathematics instruction four (grade 2) to five (grade 4) times a week.

### Materials

#### Non-verbal Cognitive Ability

Non-verbal cognitive ability was assessed with the Matrices test adopted from the Cognitive Assessment System-Version 2 (CAS-2; Naglieri et al., 2014). Children were asked to choose one piece among six alternatives to complete accurately the missing part of a graphic image that was composed of a pattern of shapes. The test was terminated when four consecutive mistakes were made. The total number of correct trials were recorded as the score of this task (maximum score = 44). Cronbach’s alpha reliability coefficient in the reliability in the CAS-S manual was 0.91 and 0.88, in grades 2 and 4, respectively (CAS-2; Naglieri et al., 2014).

#### Attention

Attention was assessed with the Expressive Attention test adopted from the CAS-2 (Naglieri et al., 2014). Children were presented with a pattern of words in a mixed variety of colors; second-graders were presented with only three colors (*red, blue*, and *yellow*) and fourth-graders were presented with four colors (*red, green, blue*, and *yellow*). They were asked to state the color of the ink of those words but not reading of the words as fast and accurate as possible. For example, the correct answer of a Chinese word “yellow” printed in red color is red. There were eight items for demonstration, eight items for practice, followed by a total of 40 words on one page for testing. Recording of time use started when children said the first color. The total number of correct answers and the time taken to state all of the words were recorded. The score of each child was calculated using the formula: 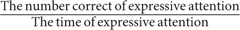. Split-half reliability coefficient in our sample was 0.95 and 0.91, in grades 2 and 4, respectively. Split-half reliability was calculated by first dividing the data into two groups using the parity grouping method and then correlating the two groups (Johnson and Penny, 2005).

#### N-back Working Memory

The N-back working memory task was adopted from the work of Kirchner (1958) and was implemented using E-prime 2.0, Psychology Software Tools, on a desktop computer. Stimuli included three solid black geometrical shapes ●(circle), ■(square) and ▲(triangle) that were randomly shown inside one of the squares of a 3 × 3 matrix. Children were asked to perform 1-back, where *n* = 1, as 2-back would be difficult for young children and reduce the test reliability (Pelegrina et al., 2015). To perform the task, children had to decide whether the geometric shape and position currently shown was the same as the one previously shown. If the shape and position of the target stimulus were both consistent with the one shown before, children were instructed to press button “A” on a laptop computer within a 3500 ms time limit, or they were instructed to press button “L.” The geometrical shapes were shown 1000 ms, and the inter-stimulus interval was 3500 ms. Children should press the button within 4500 ms, or a miss response would be recorded. Each child was given eight practice trials to familiarize the task. There were a total of 24 items in the task. The total score was the proportion of correct answers, converted from percentages (i.e., maximum score = 1). Split-half reliability in our sample was 0.85 and 0.87, in grades 2 and 4, respectively.

#### Number Line Estimation

The number line estimation task was adopted from Siegler and Opfer (2003) and was implemented on a rectangular (21.8 cm × 13.5 cm) pad. The materials included a 15-cm line with one end marked with 0 and the other end marked with 100 (i.e., min = 0, max = 100) and 28 numbers: 3, 4, 6, 8, 12, 14, 17, 18, 20, 21, 24, 25, 29, 33, 39, 42, 48, 50, 52, 57, 61, 64, 72, 79, 81, 84, 90, and 96. The number 20 and 50 were used for practice. Each child was given 26 trials to do number line estimation. In the number-to-position (NP) version of the task, which was performed in this study, children were asked to make numerical estimations by putting a single mark on each line (from 1 to 100) to indicate the location of a number presented on the number line. For example, they were given a number, say 20, and had to put a single mark on the line to indicate the location of 20. We calculated each child’s percent absolute error using the formula: 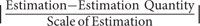. For example, if a child was asked to estimate the location of 20 on a 0–100 number line and placed the mark at the location that corresponded to 25, the percent absolute error would be 5% [(25 – 20)/100], i.e., a score of 0.05 for reflecting number line estimation errors. The lower the score, the better the number line estimation ability. Cronbach’s alpha reliability coefficient in our sample was 0.81 and 0.70, in grades 2 and 4, respectively.

#### Mathematical Skills

Two measures of mathematical skills were administered: calculation fluency and math problem-solving. Calculation fluency was adopted from WIAT-III (Wechsler Individual Achievement Test-Third Edition; Wechsler, 2009) and included two subtests: addition fluency (e.g., 2 + 1) and subtraction fluency (e.g., 7 – 1). Each subtest has 48 items, and children were asked to solve as many items as possible within a 60-s time limit. The total score was the total number of correct additions, subtractions and multiplication (max = 144). In our sample, split-half reliability coefficient was 0.88 in grade 2, and 0.93 in grade 4. Math problem-solving was also adopted from WIAT-III (Wechsler, 2009), and children were asked to solve one-on-one mathematical problems. The test was terminated when four consecutive errors were made. The total score was the total number of problems correctly solved (max = 72). Cronbach’s alpha reliability coefficient in our sample was 0.82 and 0.85, in grades 2 and 4, respectively. Split-half reliability was calculated by first dividing the data into two groups using the parity grouping method and then correlating the two groups (Johnson and Penny, 2005).

### Procedures

All children underwent two testing sessions, Session A, followed by Session B. In Session A, we administered calculation fluency in a group setting, which included a 60-s time limit for each subtest. Tests took place in a quiet room at school and were conducted by two graduate students who received extensive training on how to implement the tasks. Session B was an individual session where each child met one-on-one with the experimenter for about 60 min. The session consisted of five tests which were math problem-solving, non-verbal matrices, expressive attention, N-back, and number line estimation. The sessions were not counter-balanced or separated across children for two reasons. First, the test time was unified by the school to fit teaching schedules, and second, the students could exchange answers after class if they were assigned into groups. All test results were cross-checked for scoring accuracy, with an interrater reliability of 0.99.

### Data Analysis

Data was initially checked for outliers and skewness. Pearson correlation coefficients were computed between number line estimation, mathematical skills and cognitive measures. To examine the predictive value of number line estimation on mathematical skills, a series of hierarchical regression analyses were performed separately for each of the two grades (grade 2 and grade 4) and each of the two outcome measure (calculation fluency and math problem-solving). The predictor variables were entered into the regression equation in the following order: (1) age (in months) and non-verbal matrices, (2) attention and working memory, and (3) number line estimation. Number line estimation was the last factor to enter into the regression model; this would allow us to examine the effect of number line estimation on each of the two outcome variables after controlling for age and other cognitive variables.

## Results

### Preliminary Data Analyses

Preliminary inspection of the data indicated that both skewness and kurtosis were within acceptable levels. Descriptive statistics for all the measures used in the study are presented in **Table 1**. Independent sample *t*-test indicated that grade 4 performed better than grade 2 on math problem-solving (*t* = -11.30, *p* < 0.001), calculation fluency (*t* = -15.15, *p* < 0.001), non-verbal matrices (*t* = -2.43, *p* < 0.05), n-back (*t* = -3.30, *p* < 0.01), and number line estimation (*t* = 2.21, *p* < 0.05).

**Table 1:** Descriptive statistics for all the variables in the study.

	Grade 2				Grade 4				*t*
	*M*	*SD*	Min.	Max.	*M*	*SD*	Min.	Max.	
Math problem-solving	42.73	5.08	31.00	58.00	50.63	3.32	45.00	64.00	–11.30^∗∗∗^
Calculation fluency	65.69	13.69	39.00	98.00	104.32	17.25	71.00	136.00	–15.15^∗∗∗^
Non-verbal matrices	24.56	5.62	3.00	37.00	26.83	5.78	16.00	39.00	–2.43^∗^
Expressive attention	1.48	0.32	0.93	2.50	0.75	0.19	0.22	1.31	–
N-back	0.64	0.23	0.04	1.00	0.75	0.18	0.38	1.00	–3.30^∗∗^
Number line estimation	0.07	0.03	0.02	0.21	0.06	0.02	0.02	0.13	2.21^∗^

*^∗^*p* < 0.05; ^∗∗^*p* < 0.01; ^∗∗∗^*p* < 0.001. The materials of expressive attention were different in grade 2 and grade 4.*

### Correlations between the Measures

**Tables 2, 3** show the results of the correlation analysis performed for grades 2 and 4, respectively. In grade 2, number line estimation correlated significantly with mathematical skills (*r* = -0.27, *p* < 0.05 for calculation fluency; *r* = -0.52, *p* < 0.01 for math problem-solving), *n*-back working memory (*r* = -0.31, *p* < 0.01), and non-verbal matrices (*r* = -0.35, *p* < 0.01). In grade 4, number line estimation correlated significantly with math problem-solving (*r* = -0.38, *p* < 0.01) and non-verbal matrices (*r* = -0.32, *p* < 0.01), but not with calculation fluency, attention, and *n*-back working memory.

**Table 2:** Correlations between the measures used in our study for grade 2.

		1	2	3	4	5	6
Grade 2	1. Math problem-solving	–					
	2. Calculation fluency	0.24^∗^	–				
	3. Non-verbal matrices	0.26^∗^	–0.01	–			
	4. Expressive attention	0.30^∗∗^	0.50^∗∗^	0.09	–		
	5. N-back	0.40^∗∗^	0.23^∗^	0.32^∗∗^	0.19	–	
	6. Number line estimation	–0.52^∗∗^	–0.27^∗^	–0.35^∗∗^	–0.13	–0.31^∗∗^	–

*^∗^*p* < 0.05; ^∗∗^*p* < 0.01.*

**Table 3:** Correlations between the measures used in our study for grade 4.

		1	2	3	4	5	6
Grade 4	1. Math problem-solving	–					
	2. Calculation fluency	0.37^∗∗^	–				
	3. Non-verbal matrices	0.30^∗^	–0.08	–			
	4. Expressive attention	0.16	0.39^∗∗^	0.08	–		
	5. N-back	0.02	0.13	0.14	<0.01	–	
	6. Number line estimation	–0.38^∗∗^	–0.13	–0.32^∗∗^	–0.06	–0.23	–

*^∗^*p* < 0.05; ^∗∗^*p* < 0.01.*

### Results of Regression Analyses

**Table 4** shows the standardized beta coefficients, significance levels, and *R*^2^ changes of the regression models in each grade. The regression analyses indicated that in grade 2, number line estimation accounted for unique variance in math problem-solving (12.0%) and calculation fluency (4.0%) after controlling for age, non-verbal matrices, attention, and working memory. In grade 4, number line estimation accounted for unique variance in math problem-solving (9.0%) but not in calculation fluency after controlling for age, non-verbal matrices, attention, and working memory.

**Table 4:** Results of hierarchical regression analyses predicting calculation fluency and math problem-solving in grades 2 and 4.

		Grade 2				Grade 4			
		Calculation fluency		Math problem-solving		Calculation fluency		Math problem-solving	
Step		β	Δ*R^2^*	β	Δ*R^2^*	β	Δ*R^2^*	β	Δ*R^2^*
1.	Age	0.143	0.02	0.116	0.04	0.014	0.01	0.082	0.10^∗^
	Non-verbal matrices	–0.015		0.156		–0.083		0.302^∗^	
2.	Expressive attention	0.496^∗∗∗^	0.26^∗∗∗^	0.259^∗^	0.18^∗∗^	0.401^∗∗^	0.18^∗∗^	0.130	0.02
	N-back	0.174		0.340^∗∗^		0.158		–0.042	
3.	Number line estimation	–0.220^∗^	0.04^∗^	–0.370^∗∗^	0.12^∗∗^	–0.140	0.02	–0.318^∗^	0.09^∗∗^

*^∗^*p* < 0.05; ^∗∗^*p* < 0.01; ^∗∗∗^*p* < 0.001.*

## Discussion

The primary goal of this study was to examine the role of number line estimation on two mathematical skills (calculation fluency and math problem-solving) in children from grade 2 and grade 4. The results showed that there were significant differences in math problem solving, calculation fluency, cognitive processing abilities and number line estimation between grade 2 and grade 4 students. Grade 4 students were better than grade 2 in math problem solving, calculation fluency, non-verbal matrices, N-back and number line estimation. With the growth of age and knowledge, grade 4 students were better in analogical reasoning skills and cognitive ability which math achievement and number line estimation require (Sullivan and Barner, 2014). Booth and Siegler’s (2006) observation found that grade 4 students did better in number line estimation than grade 2 students.

In grade 2, the results of correlation analysis showed that there were significant positive correlations between math problem solving and cognitive processing abilities, number line estimation. There were significant positive correlation between calculation fluency and attention, N-back, number line estimation. In grade 4, math problem solving was significantly related to the non-verbal cognitive abilities and number line estimation, and calculation fluency was significantly related to the attention. The results of regression showed that attention significantly predicted the calculation fluency both in grade 2 and grade 4. In grade 2, attention, number line estimation and working memory can significantly predict math problem solving. It is consistent with the existing results (Swanson, 2011; Anobile et al., 2013; Traff, 2013). In grade 4, non-verbal cognitive abilities and number line estimation could predict math problem solving. The results also showed that number line estimation had a different effect in different mathematical skills after controlling the effects of age, non-verbal cognitive abilities, attention, and working memory. This reinforces the argument that number line estimation is important for mathematical skills (Siegler and Booth, 2004; Booth and Siegler, 2006; Halberda et al., 2008; Schneider et al., 2009; Cohen and Sarnecka, 2014).

On the one hand, we found that number line estimation accounted for unique variance in math problem-solving both in grade 2 and grade 4 after controlling the effects of age, non-verbal cognitive ability, attention, and working memory. It suggested that number line estimation was closely related to math problem-solving in different grades, especially in grade 2. This is in line with the general findings of several previous studies in which number line estimation was a potent concurrent predictor of mathematical achievement in young children (Siegler and Booth, 2004; Booth and Siegler, 2006; Schneider et al., 2009). Prior research demonstrated that both general cognitive abilities and number abilities were foundational abilities for learning mathematics, regardless of another types of abilities (Traff, 2013). Fuchs et al. (2010a,b) also found that the development of word problem-solving skills in children was uniquely predicted by general cognitive abilities and number abilities.

On the other hand, we also found that number line estimation accounted for calculation fluency at grade 2 after controlling for the effects of age, non-verbal cognitive ability, attention, and working memory. However, the results revealed that number line estimation was not a significant predictor of calculation fluency in grade 4. Our interpretation is that calculation fluency is the most important goal during the early school years. Problem-solving is admittedly a more complex mathematical task than calculation fluency and requires children to invest more effort to complete the task correctly (see Georgiou et al., 2010). Older children would focus more on complex mathematical skills. It is consistence with Traff (2013) that number abilities contributed to children’s arithmetic fact retrieval skills and word problem-solving beyond the contribution of age, reading, and general cognitive abilities. Another reason is that Chinese children start learning additions and subtractions earlier than other mathematical tasks, and with the increase of grade, they have better skill in calculating, so that the addition and subtraction of the following can be achieved in the process of automatic processes which require less cognitive resources. Thus, the prediction of calculation fluency showed up only in young children, as older children were able to complete the task with smaller effort.

We also found that attention was important to calculation fluency in both grade 2 and grade 4, whereas it was important to math problem-solving in grade 2, but not in grade 4. Attention and N-back together accounted for 18.0% of variance in math problem-solving and 26.0% of variance in calculation fluency after controlling for age, non-verbal matrices in grade 2. Attention and N-back together accounted for 18.0% of variance in calculation fluency, but not in math problem-solving after controlling for age, non-verbal matrices in grade 4.

The limitation of the study was worth mentioning. Although our sample size was adequate for the type of analysis we performed, grade range was relatively narrow. This might contribute to the limitation of understanding the effect of number line estimation in different grade after controlling for the effects of age, non-verbal cognitive ability, attention, and working memory. Nevertheless, it would be an important question for future studies.

To conclude, our findings showed that number line estimation might provide strong support for mathematical skills (e.g., math problem-solving) in grade 2 and grade 4 after controlling the effects of age, non-verbal cognitive ability, attention, and working memory. It showed that number line estimation played an important role in complex mathematical ability and simple mathematical tasks in lower grade children. This led to an important implication that number line estimation could boost children’s mathematics performance in different grade, particularly in lower grades.

## Ethics Statement

The study was approved by the Ethics Committee of Shanghai Normal University. Participation was completely voluntary and provided written informed consent.

## Author Contributions

DC conducted the study, designed of the work, recruited participants, analyzed and interpreted the data, and wrote and revised the manuscript. MZ recruited participants, collected data, analyzed and interpreted the data, and wrote the draft of the manuscript and revised the draft of the manuscript. AL helped writing the draft of the manuscript, provided consultation and revised the draft of the manuscript.

## Conflict of Interest Statement

The authors declare that the research was conducted in the absence of any commercial or financial relationships that could be construed as a potential conflict of interest.
